# Inhibition of Histo-blood Group Antigen Binding as a Novel Strategy to Block Norovirus Infections

**DOI:** 10.1371/journal.pone.0069379

**Published:** 2013-07-19

**Authors:** Xu-Fu Zhang, Ming Tan, Monica Chhabra, Ying-Chun Dai, Jarek Meller, Xi Jiang

**Affiliations:** 1 Divisions of Infectious Diseases, Cincinnati Children’s Hospital Medical Center, Cincinnati, Ohio, United States of America; 2 School of Traditional Chinese Medicine, Southern Medical University, Guangzhou, Guangdong, China; 3 Department of Pediatrics, University of Cincinnati College of Medicine, Cincinnati, Ohio, United States of America; 4 Department of Computer Science, University of Cincinnati College of Engineering, Cincinnati, Ohio, United States of America; 5 Department of Epidemiology, School of Public Health and Tropical Medicine, Southern Medical University, Guangzhou, Guangdong, China; 6 Department of Environmental Health, University of Cincinnati College of Medicine, Cincinnati, Ohio, United States of America; Russian Academy of Sciences, Institute for Biological Instrumentation, Russian Federation

## Abstract

Noroviruses (NoVs) are the most important viral pathogens that cause epidemic acute gastroenteritis. NoVs recognize human histo-blood group antigens (HBGAs) as receptors or attachment factors. The elucidation of crystal structures of the HBGA-binding interfaces of a number of human NoVs representing different HBGA binding patterns opens a new strategy for the development of antiviral compounds against NoVs through rational drug design and computer-aided virtual screening methods. In this study, docking simulations and virtual screening were used to identify hit compounds targeting the A and B antigens binding sites on the surface of the capsid P protein of a GII.4 NoV (VA387). Following validation by re-docking of the A and B ligands, these structural models and AutoDock suite of programs were used to screen a large drug-like compound library (derived from ZINC library) for inhibitors blocking GII.4 binding to HBGAs. After screening >2 million compounds using multistage protocol, 160 hit compounds with best predicted binding affinities and representing a number of distinct chemical classes have been selected for subsequent experimental validation. Twenty of the 160 compounds were found to be able to block the VA387 P dimers binding to the A and/or B HBGAs at an IC_50_<40.0 µM, with top 5 compounds blocking the HBGA binding at an IC_50_<10.0 µM in both oligosaccharide- and saliva-based blocking assays. Interestingly, 4 of the top-5 compounds shared the basic structure of cyclopenta [a] dimethyl phenanthren, indicating a promising structural template for further improvement by rational design.

## Introduction

Noroviruses (NoVs) are a group of single-stranded, positive sense RNA viruses constituting the *Norovirus* genus in the family *Caliciviridae.* NoVs have been recognized as the most important cause of viral epidemic acute gastroenteritis affecting people of all ages [1], [2]. In the United States NoVs cause 23 million infections each year and are responsible for more than 90% of the outbreaks of viral gastroenteritis. On a worldwide basis NoVs lead to 218,000 deaths in developing countries and 1.1 million episode of pediatric gastroenteritis in developed countries annually [3]. Thus, NoV associated diseases have been a heavy burden to public healthcare. NoVs are difficult to control owing to their widespread nature and the lack of effective vaccines and antivirals.

NoVs are non-enveloped viruses that are encapsulated by an icosahedral protein capsid comprising 180 copies of the single major structural protein, the capsid protein (VP1). Based on its structural features, the capsid protein is divided into two major domains, the shell (S) and the protruding (P) domains, each forming the interior shell and the arch-like protrusions of NoV capsid, respectively. The P domain can be further divided into P1 and P2 subdomains, constituting the leg and the head of the arch-shaped P dimer, respectively [4]. The P domain plays an important role in host immune response and receptor recognition. Heterologous expression of the P domain in *E. coli* forms P dimers [4] that is structurally and functionally indistinguishable from the authentic P dimers of viruses [5]–[11], providing a simple model for study of NoV-host interaction [12]–[17]. In addition, production of P domain with end modifications can also form 24 mer P particles [12], [14], [15] and 12 mer small P particle [13], which contain 12 and 6 copies of P dimers, respectively.

NoVs recognize human histo-blood group antigens (HBGAs) as receptors or attachment factors, which play an important role in host susceptibility to NoV infection [18]–[21]. HBGAs are complex carbohydrates that are presented abundantly on the surface of mucosal epithelia of gastrointestinal track, where they may function as anchors for NoVs to initiate an infection. Human HBGAs are highly polymorphic that contain three major families, the ABO, secretor and Lewis families. Human NoVs are also highly diverse and multiple receptor binding patterns with different ABO, secretor and Lewis antigens have been described. The HBGA interacting sites have been mapped to the P domain of NoV capsid [4], [12]–[14], [22]. Further X-ray crystallography of the recombinant P dimers of a number of human NoVs representing different HBGA binding patterns in complex with different HBGA oligosaccharides has been resolved which provided valuable structural basis of the HBGA-NoV interactions [5]–[10].

The HBGA binding interfaces are located at the top of the P dimer, corresponding to the outermost surface of the capsid. The carbohydrate binding pockets involve several scattered amino acid residues in the P domain that form extensive hydrogen bond network with individual saccharides, and thus stabilizing the binding of HBGAs to the capsid protein. Structure-based mutagenesis followed by functional analyses further confirmed the observed HBGA binding sites [7], [15], [16]. This detailed structural information of NoV-HBGA interactions opens a way to a new strategy for antiviral development through Computer-Aided Drug Design (CADD), while the established biological assays of NoV P dimer-HBGA interaction provide a convenient approach for validation of hit compounds identified by CADD.

CADD is a common approach in drug discovery that typically involves following major steps: 1) construction and validation of computational models of the target protein based on its crystal structures with known functional (HBGA binding) sites; 2) virtual high throughput screening (VHTS) of a large number of chemical compounds to identify candidate inhibitors (hit compounds) that are predicted to bind to the functional site of the protein with sufficiently high affinity; and 3) validation of the candidate inhibitors through biological and biochemical assays. Further development of promising candidate inhibitors includes assessment of their toxicity, pharmacokinetic and rational re-design based on structures of individual candidates, with the goals of improving efficacy while lowering toxicity and other undesired properties.

VHTS has been widely used for candidate compound discovery due to an advantage in elimination of undesired molecules from compound libraries, so that the cost and labor can be greatly reduced in a drug discovery project. A number of public compound libraries are currently available for VHTS, including Zinc (http://zinc.docking.org/) [23], NCI [http://cactus.nci.nih.gov/download/nci/], UC Irwine Chem BD [24], and Ligand-Depot [25]. VHTS of large databases of chemical compounds has been repeatedly shown to successfully identify hit compounds that can effectively inhibit the function of a given protein [26]–[29].

In this study we report the first attempt to identify inhibitors of HBGA binding to NoVs as potential antivirals against NoVs through a CADD procedure. The crystal structures of the VA387 (a member of the predominant GII.4 NoVs) P dimer interacting with HBGA were employed to construct computer models for VHTS. After validations of the models by virtual docking simulation using the type A- and B-trisaccharides as ligands, VTHS of a large drug-like compound library was performed, resulting in 255 hit compounds. A total of 160 compounds of the compounds were further tested by biological assays and five revealed strong blocking activity on P dimer-HBGA interaction with an IC_50_<10 µM. Our results suggest that the CADD approach can facilitate the development of antivirals against human NoVs and the five highly active compounds could become a basis for developing promising drug candidates against NoVs.

## Materials and Methods

### Computer Models of P Dimers and Oligosaccharides

The two PDB files revealing the crystal structures of the P dimers of VA387 (a predominant GII.4 NoVs) in complex with A (2OBS) and B (2OBT) HBGA oligosaccharides, respectively [6], were downloaded from Protein Data Bank (PDB, http://www.pdb.org). The protein structures with removal of the HBGA ligands were used as model of P dimer, while the extracted HBGA structures were used as models of A and B trisaccharides. Preparation of the HBGA model included specifying rotatable bonds, assigning partial charges, and preparing grid boxes for docking simulations, which was performed using AutoDock package. Preparation of the P dimer model included adding polar hydrogens, checking missing atoms, assigning charges and solvation parameters. Information of HBGA oligosaccharides interacting with the amino acids that constitute the HBGA binding sites were used to set parameters for molecular docking.

### Docking Simulation

Re-docking simulations were used for validation of the structural models and docking protocols. The results obtained by docking the native ligands (A and B trisaccharides) to the P dimer were compared with the structural data on P dimer in complex with HBGAs [6]. Using AutoDock 3 package [30] we performed a series of rigid (P dimer) body simulations, followed by additional assessment of the results from “flexible ligand-flexible key binding residues of the P dimer” docking simulations. We found that rigid body docking was qualitatively consistent with flexible docking simulations, and able to reproduce experimentally observed conformations of the P dimer - HBGAs complexes. In particular, majority of over 200 different poses generated in repeated rigid body simulations were found to be in good agreement with experimental data. Grid boxes and grid densities for rigid body docking were optimized to provide sufficient accuracy and to cover the binding site(s) that might occur over the whole P dimer molecule (blind docking). Docked conformations of ligands were generated by AutoDock’s Lamarckian genetic algorithm (GA) [31]. Docking parameters used for the simulations are listed in Table 1. Docking simulations and the VTHS (see below) were conducted on the Cincinnati Children’s Hospital Medical Center’s BMI computational cluster with over 200 processing cores (at least 2.4 GHz) running 64-bit SuSE Linux operating system.

**Table 1:** Parameters used for docking simulation.

Iteration Parameters	
Translation step	2 Å
Quaternion step	50°
Torsion step	50°
Lamarckian Genetic Algorithm Parameters	
Number of Genetic Algorithm runs	255
Initial population size	300
Maximum number of energy evaluations	2.5 million
Maximum number of generations	35,000
No. of top individuals that automatically survive	1
Rate of gene mutation	0.02
Rate of crossover	0.8
Number of generations for picking worst individual	10
Number of iterations of pseudo Solis and Wets local search	300
Number of consecutive successes before changing	4
Number of consecutive failures before changing	4
Probability of performing local search on an individual	0.06
Grid Parameters	
Grid Spacing	0.375 Å
Number of grid points in x, y, and z directions	78, 50, 45

### VHTS of the Compound Library

A subset of the public compound library ZINC, consisting of 2,066,906 drug-like compounds was downloaded from the ZINC database (http://zinc.docking.org/). In the initial stage of VTHS, these compounds were subjected to the automatic molecular docking using the “rigid P dimer-flexible ligand” approach and AutoDock ver. 3. The primary screening was done using coarse-level docking with limited sampling [26], [32]. Compounds predicted to have <0.1 µM inhibition constant (Ki<10^−7^) were subsequently subjected to a secondary round of screening with improved sampling. The protocol used for secondary screening (see Table 2) involved an increased number of overall simulation runs, increased number of energy evaluation, increased size of the GA population [33], and a finer grid resolution (decreased from 0.6 to 0.375 Ang). The identified top 255 hit compounds that were predicted to bind the HBGA binding site with better affinity than that of A and B oligosaccharides were selected for experimental validation. The bound P dimer structures of VA387 (2OBS) [6] with removal of the A-trisaccharides were used as model for the screening. The docking parameters for the primary VHTS were similar to those used in molecular docking (Table 2). To reduce the docking time, trivial parallelism of VHTS was exploited by performing docking simulations for subsets of compounds on individual computing nodes using a pipeline described in Biesiada et al [32].

**Table 2:** Parameters used in the primary and secondary screening of the VHTS.

Iteration Parameters	Primary	Secondary
Translation step	2 Å	2 Å
Quaternion step	50°	50°
Torsion step	50°	50°
Lamarckian Genetic Algorithm Parameters		
Number of Genetic Algorithm runs	10	100
Initial population size	50	500
Maximum number of energy evaluations	150,000	500,000×torsion
Maximum number of generations	27,000	27,000
No. of top individuals that automatically survive	1	1
Rate of gene mutation	0.02	0.02
Rate of crossover	0.8	0.8
Number of generations for picking worst individual	10	10
Number of iterations of pseudo Solis and Wets local search	300	300
Number of consecutive successes before changing	4	4
Number of consecutive failures before changing	4	4
Probability of performing local search on an individual	0.06	0.06
Grid Parameters		
Grid Spacing	0.6 Å	0.375 Å
Grid points in x, y, and z directions	49, 32, 29	Split into two grids

### Purchases of the Hit Compounds

160 top hits (which were actually selected based on their availability from a somewhat larger initial set of 255 hit compounds obtained in virtual screening) were purchased from Molport (http://www.molport. com) supplied by Maybridg, TimTec, ChemBridge, Pharmeks, Specs, Otava, ChemDiv, InterBioScreen Ltd, Vitas-M Laboratory, Princeton Biomolecular Research and Enamine Ltd. The information including ZINC numbers and structures of the 160 chemicals were listed in Table S1 and Figure S1. All compounds were dissolved at 100 µg/ml in PBS (pH 7.4) containing 1% DMSO as stock solutions.

### Validation of Hit Compounds by NoV/HBGA Blocking Assays

Saliva-based NoV-HBGA binding assays were performed as described previously using P dimers of VA387 (GII.4) as NoV surrogates and saliva samples and/or synthetic oligosaccharides as HBGA sources [4], [34], [35]. Briefly, synthetic oligosaccharides and/or boiled saliva samples with defined HBGAs phenotype were coated on 96-well microtiter plates, after blocking with nonfat milk, P dimer of VA387 were added. The bound P dimer was detected using a guinea pig antiserum against NoVs VLPs, followed by the addition of horseradish peroxidase (HRP)-conjugated goat anti-guinea pig IgG. The bound HRP conjugates were colorized by the TMB kit (Kirkegaard & Perry Laboratories), which was read an EIA spectrum reader (Tecan). The synthetic oligosaccharide-PAA conjugates (2 µg/ml, GlycoTech Corporation, Rockville, MD) were captured to a microtiter plate through coated streptavidin (5 µg/ml) [36].

Blocking effects of the hit compounds were measured by a pre-incubation of the P dimers with the compounds at given concentrations for 30 min before the P dimers were added to the coated saliva samples or HBGA oligosaccharides in a binding assay [37]. The blocking activity of a compound was defined as its concentration yielding 50% inhibition (IC_50_) in the binding assay. IC_50_ calculation was performed using Probit regression analysis and correlation analysis between IC_50_ and K_i_ values was performed with nonparametric Spearman’s r (two-tailed) by using SPSS statistical software version 13.0 (SPSS, Chicago, IL).

### MTS Cytotoxicity Assay

This assay was performed using CellTiter 96 aqueous nonradioactive cell proliferation kits (Promega, Madison, WI) as described elsewhere [36]. Briefly, HeLa and LLC-MK2 cells were seeded at 5×10^4^ cells/ml onto a 96-well plate overnight. After an incubation with each compound at various concentrations for 3 days (the compound was added one time at the beginning of the incubation), the culture medium was replaced with fresh one with 100 µl of MTS-phenazine methosulfate/well. After a further incubation at 37°C for 2 h, the color products of MTS were measured with a plate reader at 490 nm. The cytotoxicities of individual compounds were indicated by the decrease in cellular reduction of MTS into the colored product. The 50% cytotoxic concentrations (CC_50 s_) were determined as the concentrations of the compounds that caused 50% inhibition of cell growth compared with that of control cells without a compound.

## Results

### Validation of the NoV Model and the Molecular Docking Protocol

**Figure 1:**
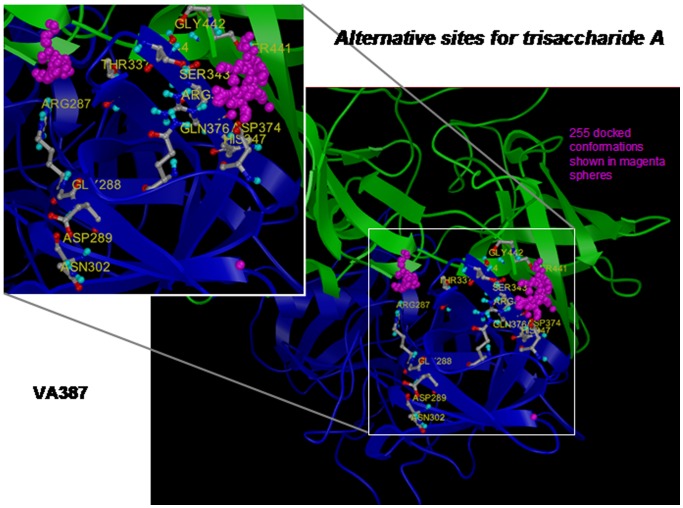
Predicted docking poses for the A trisaccharides docked to the VA387 P dimer clusters at the proved HBGA binding site. The majority of the predicted poses (each represented by a magenta ball) docked to the experimentally resolved HBGA binding site that was formed by Ser343, Arg345, His347, Asp374, Gln376, Ser441 and Gly442 (circled by a yellow dashed line). Some of the poses docked to a nearby site that was previously suggested as an alternative binding site for HBGA (Tan et al., 2003). The structure of the P dimer of VA387 (2OBS) is shown using ribbon model, each monomer in green and blue, respectively. The amino acids that constitute the experimentally mapped HBGA binding site were shown using stick model and circled by a yellow dashed line, while amino acids forming the nearby site were also shown in stick model. The squared region containing the HBGA binding site is enlarged in the up-left panel.

The crystal structures of NoV P dimers of VA387 (GII.4) with HBGA oligosaccharides (2OBS and 2OBT) [6] were used to build target structures for molecular docking and VTHS of the ZINC compound library. The models were first employed for validation by re-docking simulations using a type A trisaccharide as a ligand through software AutoDock 3 [30]. As shown in Figure 1, the vast majority of multiple poses of the A trisaccharide obtained in repeated simulations docked well to the experimentally mapped HBGA binding site of VA387 that was formed by seven amino acids: Ser343, Arg345, His347, Asp374, Gln376, Ser441 and Gly442. It was noted that some conformations of the A trisaccharide docked to an undefined nearby site. The predicted inhibition constants (K_i_) were relatively low (with the best predicted K_i_ of about 1.6 µM), reflecting the nature of trisaccharide-protein interactions. Similar results were obtained when docking simulations were performed using the type B trisaccharide as a ligand (data not shown). Thus, these data are consistent with the crystal structures of the VA387 P dimer and provide validation of our computational models and molecular docking protocols.

### VTHS and Laboratory Validation of Drug-like Compounds Against NoV Binding to HBGAs

**Figure 2:**
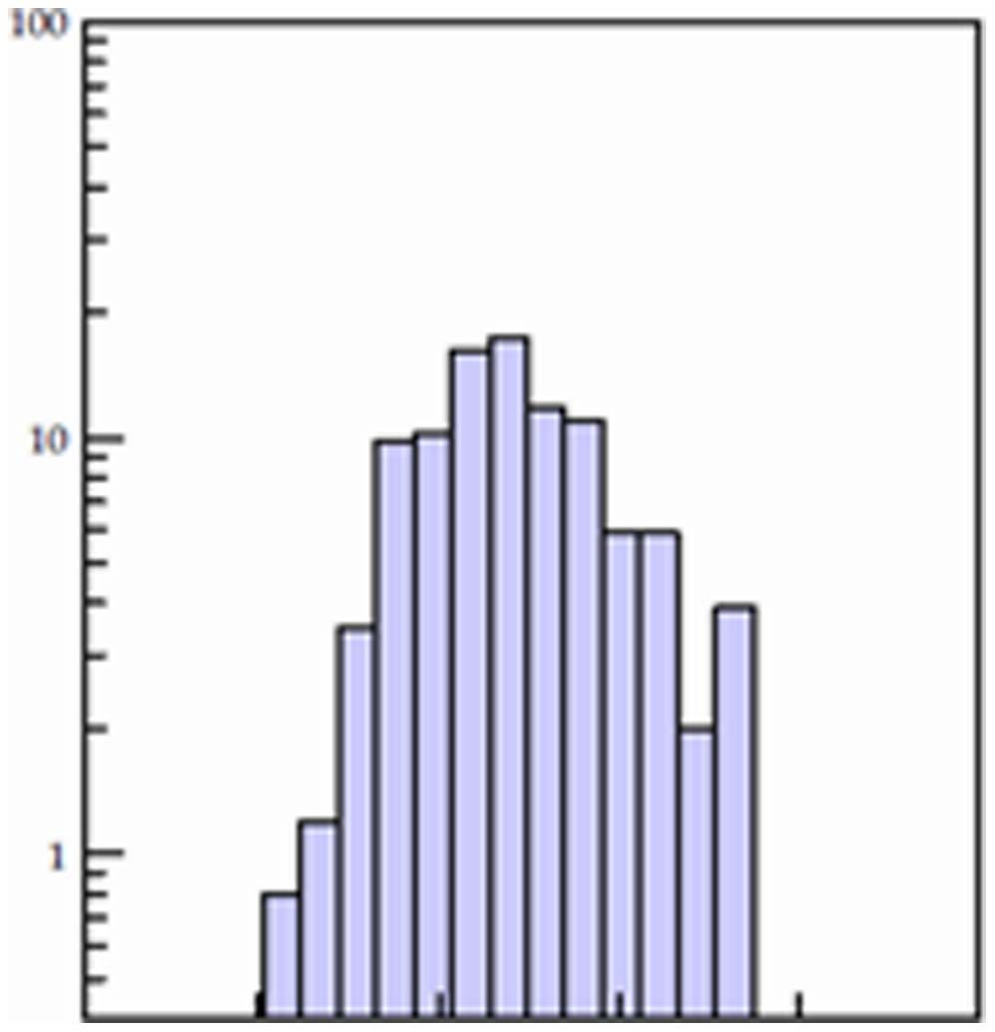
Distribution of the lowest Ki values of the top 255 compounds docked at the HBGA binding sites of the VA387 P dimer.

**Figure 3:**
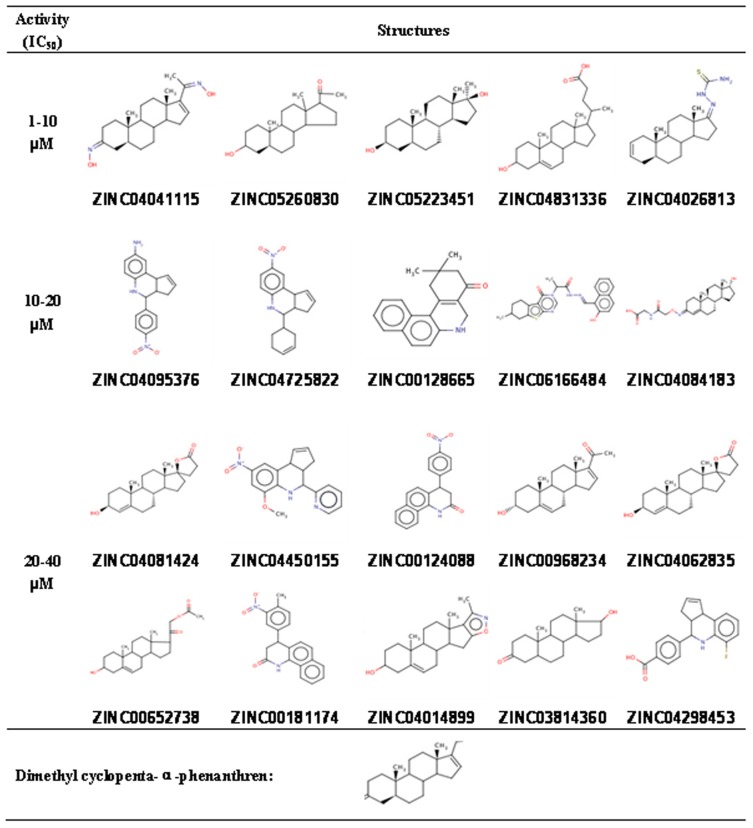
Structures of the top 20 hit compounds against binding of VA387 to the A and B saliva.

**Figure 4:**
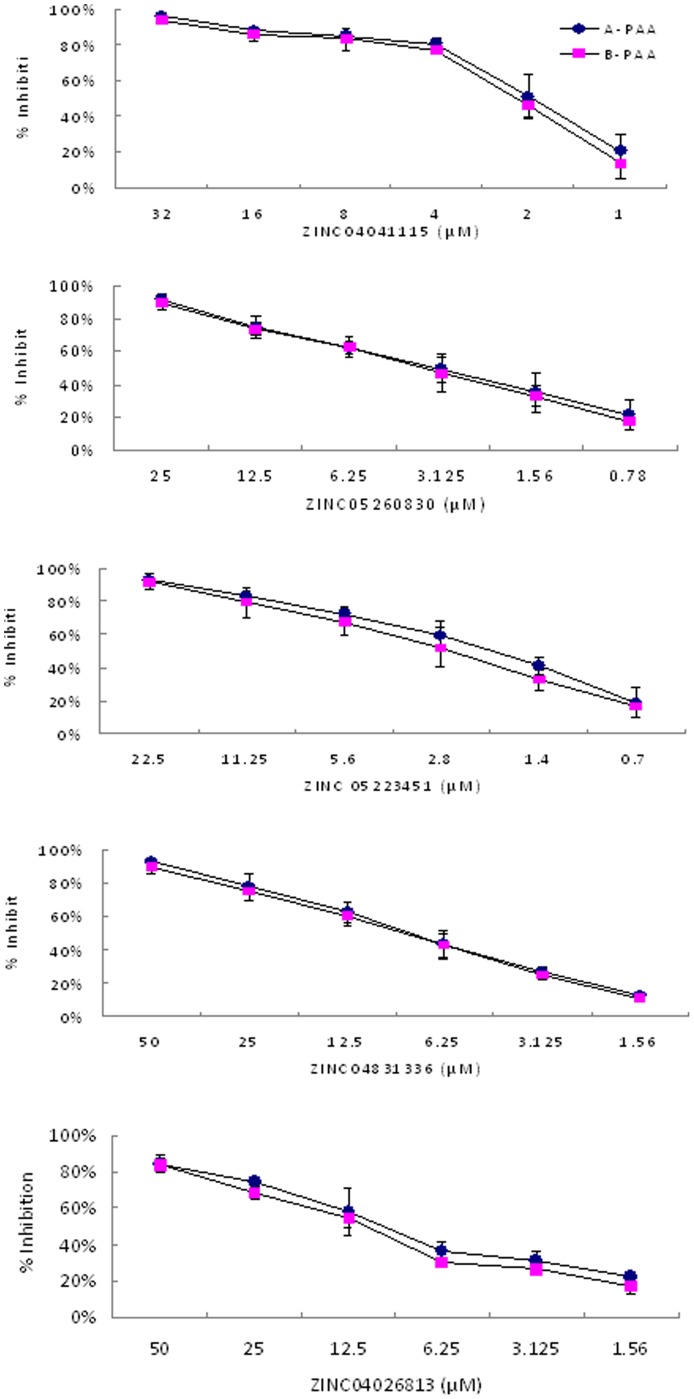
Validation and titration of inhibitory activities of the top 5 hit compounds to HBGA oligosaccharide-PAA conjugates. All compounds revealed significantly blocking activities against VA387 binding to the oligosaccharide-PAA conjugated A and B. The concentrations of the compounds used in the assays were adjusted according to their blocking activities in the type A and type B saliva screening. The IC_50_ concentrations of these 5 compounds were lower than 10 µM. Triplicate tests for each compound were performed, and the mean reduction of binding activity was presented.

Multistage screening of ∼ 2.07 million compounds from the ZINC drug-like library has resulted in identification of 255 hit compounds with predicted inhibition constants Ki values less than 100 µM against VA387 binding to the A and/or B trisaccharides (Figure 2). A total of 160 compounds from the 255 compounds were purchased from several different companies based on their availability (see Materials and Methods) and tested by saliva-based blocking assays using VA387 P dimers as NoV surrogates and type A and B saliva samples as HBGA sources. Twenty compounds (12.5%) exhibited >50% inhibitory effects on the P dimer-saliva interactions at a concentration <40 µM (Figure 3). The specificities of the top 20 compounds were further studied by both saliva- and synthesized HBGA oligosaccharide-based blocking assays. Five of the 20 compounds showed strong inhibitions with IC_50 s_ <10 µM; five others revealed good inhibitions with IC_50 s_ ranging from 10 to 20 µM, while the remaining ten compounds exhibited moderate inhibitions with IC_50 s_ ranging from 20–40 µM (Table 3). All of the top five strongest inhibitors (ZINC04041115, ZINC05260830, ZINC05223451, ZINC04831336 and ZINC04026813) revealed similar levels of inhibition in a dose-dependent manner against the VA387 P dimer binding to the synthetic A and B oligosaccharides (Figure 4). A marginal correlation between the IC_50_ values from the block assays and the K_i_ values from the docking simulation of the 20 top-list compounds were observed (Spearman *r* = 0.561, *p* = 0.01, data not shown).

**Table 3:** The basic features of the 20 most inhibitory lead-like compounds.

ZINC-codes	MW(Da)	MolecularFormula	Ki value(µM)a	IC50 (µM)b	
				Saliva A	Saliva B
ZINC04041115	344.49	C21H32N2O2	0.64	2.38±0.15	2.54±0.21
ZINC05260830	318.49	C21H34O2	1.25	2.90±0.33	2.93±0.18
ZINC05223451	306.48	C20H34O2	1.14	3.37±0.13	3.39±0.24
ZINC04831336	374.56	C24H38O3	0.36	7.63±0.27	7.65±0.33
ZINC04026813	345.55	C20H31N3S	0.16	8.70±1.03	8.97±0.63
ZINC04095376	307.35	C18H17N3O2	3.25	12.6±0.87	12.8±0.64
ZINC04725822	296.36	C18H20N2O2	1.65	13.2±1.04	13.1±0.87
ZINC00128665	277.36	C19H19NO	1.58	14.1±0.53	14.9±0.71
ZINC06166484	460.55	C25H24N4O3S	1.16	17.0±1.06	16.8±0.87
ZINC04084183	418.53	C23H34N2O5	0.42	18.7±0.35	18.6±0.62
ZINC04081424	344.49	C22H32O3	0.35	22.7±1.36	23.7±1.28
ZINC04450155	323.35	C18H17N3O3	7.77	24.2±0.74	24.3±0.87
ZINC00124088	318.33	C19H14N2O3	13.2	24.5±0.98	26.3±1.24
ZINC00968234	314.46	C21H30O2	0.92	24.8±2.02	25.1±1.32
ZINC04062835	274.4	C18H26O2	2.49	28.5±1.54	29.4±1.24
ZINC00652738	374.51	C23H34O4	1.67	30.9±1.04	30.1±1.35
ZINC00181174	332.35	C20H16N2O3	3.85	33.5±0.68	33.7±1.27
ZINC04014899	327.46	C21H29NO2	1.56	33.9±1.67	34.5±1.93
ZINC03814360	290.44	C19H30O2	2.20	36.9±1.47	35.7±1.06
ZINC04298453	309.33	C19H16FNO2	22.0	38.0±2.01	39.4±1.53

a determined by docking;

b determined by blocking assays; the data were indicated by mean ± standard deviation.

### Structure Comparisons of the Top 20 Hit Compounds

The chemical structure of the 20 top-list compounds are shown in Figure 3 according to their IC_50 s_ in blocking the binding of NoV P dimer to HBGAs. Interestingly, over a half of them share the basic structure of cyclopenta [a] dimethyl phenanthren. Particularly, four of the five most potent inhibitors share this common structure, suggesting that the cyclopenta [a] phenanthren with dimethyl may represent a promising class of compounds for further refinement.

### The Cytotoxicity of Top 5 Hit Compounds

This was tested in human cervical carcinoma cells (HeLa) and rhesus monkey kidney epithelial cells (LLC-MK2). The resulting CC_50 s_ were 203.2 (ZINC04041115), 266.8 (ZINC05260830), 170.6 (ZINC05223451), 225 (ZINC04831336) and 212.4 (ZINC04026813) µM, respectively.

## Discussion

Currently there is no effective intervention available against NoV gastroenteritis. We described in this study a search for small compounds to inhibit NoV binding to HBGAs as potential antivirals against NoVs. The computer-aided drug development (CADD) approach was used. Computational models of NoV P dimers and their ligands (A and B trisaccharides) were constructed based on available crystal structures [6]. After validation of the models and docking protocol, over two million compounds in the ZINC library were screened by docking simulations and virtual screening. A total of 255 hit candidates have been identified, among which 160 were further tested by HBGA blocking assays. Twenty compounds (12.5%) exhibited >50% inhibitory effects (IC_50_) at concentrations below 40 µM, in which five have an IC_50_<10 µM. This study suggested the CADD is a useful approach for antiviral development against NoVs.

Virtual screening based on validated computer models allows elimination of undesired molecules from large compound libraries, thus greatly reducing the cost and time of drug discovery process. In our case, experimentally screening of a library with more than two million compounds is impossible due to the huge workloads. However, the VHTS approach resulted in only 255 hit candidates with theoretical binding affinity higher than the native HBGA ligands within a few weeks of computation time, which greatly facilitated our study to the next step of biological assays for validation. Nevertheless, computer-aided virtual screening also has shortcomings. For example, some active compounds or structures may be screened off by virtual screening and thus may not be tested. This limitation should be kept in mind when the CADD is used.

We identified five compounds with high affinities (IC_50 _s <10 µM) to the HBGA binding sites of GII.4 NoVs. Further development of effective antivirals against NoVs based on these top five compounds is possible. However, we also are aware that the assays used in our study were based on monovalent interaction which may not represent the natural interactions between NoVs and human HBGAs which are multivalent. Thus, future studies to develop polyvalent inhibitors by conjugation of the hit compounds with large molecular back-bones for higher efficient blocking against NoVs are necessary. In addition, in the measurement of the cytotoxicity (CC_50_) of the top 5 hit compounds, the compounds were added to the cell media at the beginning of the three-day incubation without further monitoring their stability. This experiment needs to be improved in our future studies. Furthermore, we noticed that all the five top candidates have the common structures of steroid pregnanolone. This needs to be considered in our future studies to avoid potential side effects of hormone activities of the candidates as well as their derivatives.

In our docking simulations using the A or B trisaccharide as ligands, both ligands could be docked to an undefined nearby site around Arg 289 (Figure 1). This type of extra docking site is understandable owing to the protein/carbohydrate interaction nature that is involved in conformational network interactions. It is known that a single amino acid difference can significantly change the outcome of HBGA interaction in a mutagenesis studies of the HBGA binding interface of NoVs [4], [15], [16], [38] [7], [8], [16], [39]. The receptor binding sites of human NoVs are known highly conserved and no extra binding site was found in any known crystal structures of NoVs. Thus, the extra docking site was ignored in our study.

The shared structures of cyclopenta [a] dimethyl phenanthren among most hit compounds suggest an important lead class of compounds for future development, although such structures also shared with the steroid pregnanolone. The low toxicity of all the top five compounds to HeLa and LLC-MK2 cells further suggested this basic structure as a promising lead class. Thus, future studies to define the major functional domains for the blocking activities against NoVs as well as to avoid or reduce the potential hormone activities of pregnanolone of these compounds are critical for our rational design for maximal blocking activities with least toxicity or side effects.

Human NoVs are diverse in recognizing different HBGAs. However, the receptor binding interfaces of different NoVs are structurally conserved among strains within the two major genogroups of human NoVs. This suggests that the CADD approach described in this study may be extended to other NoVs recognizing different HBGAs because of a potential common mechanism of virus/HBGA interaction for all human NoVs. In addition to the GII.4 NoVs that were explored in this study, the crystal structures of several other NoVs with different genetic backgrounds and HBGA binding profiles have also been determined, including the GI.1 Norwalk virus [5], [8], GI.2 FUV258 [11], GII.9 VA207 [7], GII.10 Vietnam 026 [10] and GII.12 Hiro [10]. Future studies to develop antivirals for other NoVs using the same CADD procedures described in this study may be fruitful, because a good correlation between the IC_50 s_ in the blocking assays and the K_i_ values by the docking simulations has been found which support the VHTS as a useful approach for antiviral development against NoVs.

## Supporting Information

Figure S1 **The structures of the 160 hit compounds that were purchased for validation by blocking assay.**
(DOC)

Table S1 (DOCX)
