# Supplementary material for: Inhibition of Histo-blood Group Antigen Binding as a Novel Strategy to Block Norovirus Infections
Source: PLoS One. 2013 Jul 19;8(7):e69379. doi: 10.1371/journal.pone.0069379 (PMC3716607; doi:10.1371/journal.pone.0069379)
Supplement: Table S1 — (DOCX) [file pone.0069379.s002.docx]

| No. | ZINC # | No. | ZINC # | No. | ZINC # | No. | ZINC # |
| --- | --- | --- | --- | --- | --- | --- | --- |
| 1 | ZINC04369996 | 41 | ZINC04387614 | 81 | ZINC04004547 | 121 | ZINC02117024 |
| 2 | ZINC04369983 | 42 | ZINC00480643 | 82 | ZINC01428385 | 122 | ZINC02124329 |
| 3 | ZINC04289111 | 43 | ZINC00135920 | 83 | ZINC03123167 | 123 | ZINC01780068 |
| 4 | ZINC04334984 | 44 | ZINC06166484 | 84 | ZINC03878520 | 124 | ZINC04026813 |
| 5 | ZINC00173967 | 45 | ZINC04293011 | 85 | ZINC0467627 | 125 | ZINC13147458 |
| 6 | ZINC04369755 | 46 | ZINC04168293 | 86 | ZINC02197329 | 126 | ZINC04731265 |
| 7 | ZINC04950935 | 47 | ZINC02722961 | 87 | ZINC04697090 | 127 | ZINC04044743 |
| 8 | ZINC00119434 | 48 | ZINC02941133 | 88 | ZINC04014899 | 128 | ZINC04140397 |
| 9 | ZINC01439297 | 49 | ZINC06721367 | 89 | ZINC02350003 | 129 | ZINC05016671 |
| 10 | ZINC05223451 | 50 | ZINC02077712 | 90 | ZINC04676626 | 130 | ZINC01698345 |
| 11 | ZINC00128677 | 51 | ZINC02127317 | 91 | ZINC03073677 | 131 | ZINC04140392 |
| 12 | ZINC03831599 | 52 | ZINC05260830 | 92 | ZINC00645731 | 132 | ZINC18188183 |
| 13 | ZINC00128665 | 53 | ZINC03904495 | 93 | ZINC00181174 | 133 | ZINC00307237 |
| 14 | ZINC04831336 | 54 | ZINC06779985 | 94 | ZINC04181716 | 134 | ZINC03351147 |
| 15 | ZINC01131346 | 55 | ZINC03871563 | 95 | ZINC00052555 | 135 | ZINC03226596 |
| 16 | ZINC04833316 | 56 | ZINC04029585 | 96 | ZINC04576425 | 136 | ZINC03391699 |
| 17 | ZINC00202070 | 57 | ZINC04084183 | 97 | ZINC02110792 | 137 | ZINC04321822 |
| 18 | ZINC04718490 | 58 | ZINC04081424 | 98 | ZINC04329534 | 138 | ZINC03886431 |
| 19 | ZINC04982434 | 59 | ZINC04041115 | 99 | ZINC00124088 | 139 | ZINC00136236 |
| 20 | ZINC04450155 | 60 | ZINC04064920 | 100 | ZINC00094792 | 140 | ZINC04554348 |
| 21 | ZINC04983852 | 61 | ZINC04029396 | 101 | ZINC00339399 | 141 | ZINC02113682 |
| 22 | ZINC02999226 | 62 | ZINC04082577 | 102 | ZINC03814360 | 142 | ZINC18268204 |
| 23 | ZINC02873243 | 63 | ZINC04062835 | 103 | ZINC00649517 | 143 | ZINC02287313 |
| 24 | ZINC04981456 | 64 | ZINC00968234 | 104 | ZINC05748824 | 144 | ZINC04554354 |
| 25 | ZINC05023522 | 65 | ZINC01823138 | 105 | ZINC00644369 | 145 | ZINC02083349 |
| 26 | ZINC04818957 | 66 | ZINC04082451 | 106 | ZINC04546574 | 146 | ZINC04721062 |
| 27 | ZINC04081186 | 67 | ZINC00752133 | 107 | ZINC00832965 | 147 | ZINC04554342 |
| 28 | ZINC04818889 | 68 | ZINC04025453 | 108 | ZINC04104841 | 148 | ZINC00094443 |
| 29 | ZINC04298453 | 69 | ZINC04026581 | 109 | ZINC04320367 | 149 | ZINC16038467 |
| 30 | ZINC04725822 | 70 | ZINC04370120 | 110 | ZINC04151424 | 150 | ZINC02350512 |
| 31 | ZINC00197884 | 71 | ZINC04557061 | 111 | ZINC03904176 | 151 | ZINC01060583 |
| 32 | ZINC02438686 | 72 | ZINC05156742 | 112 | ZINC12414169 | 152 | ZINC01896793 |
| 33 | ZINC04817083 | 73 | ZINC04412768 | 113 | ZINC04028983 | 153 | ZINC04601352 |
| 34 | ZINC04073908 | 74 | ZINC02124139 | 114 | ZINC02117903 | 154 | ZINC09009480 |
| 35 | ZINC01745238 | 75 | ZINC05196295 | 115 | ZINC04183327 | 155 | ZINC09089608 |
| 36 | ZINC04095376 | 76 | ZINC04708275 | 116 | ZINC05529848 | 156 | ZINC06344045 |
| 37 | ZINC04004607 | 77 | ZINC00677962 | 117 | ZINC04027511 | 157 | ZINC04556826 |
| 38 | ZINC01019606 | 78 | ZINC00652738 | 118 | ZINC05383398 | 158 | ZINC04558234 |
| 39 | ZINC04666938 | 79 | ZINC01091786 | 119 | ZINC04027729 | 159 | ZINC00264290 |
| 40 | ZINC04651589 | 80 | ZINC04544325 | 120 | ZINC02132404 | 160 | ZINC03830639 |

Table S1: The ZINC numbers of the 160 lead-like compounds that were purchased for further characterization.
